# Tear-Based Ocular Wearable Biosensors for Human Health Monitoring

**DOI:** 10.3390/bios14100483

**Published:** 2024-10-08

**Authors:** Arunima Rajan, Jithin Vishnu, Balakrishnan Shankar

**Affiliations:** 1 Centre for Flexible Electronics and Advanced Materials, Amrita Vishwa Vidyapeetham, Amritapuri 690525, India or arunimarajan@am.amrita.edu (A.R.); or jithinv@am.amrita.edu (J.V.); 2 Department of Mechanical Engineering, Amrita Vishwa Vidyapeetham, Amritapuri 690525, India

**Keywords:** biosensors, wearable, healthcare, ocular, tear, biomarkers

## Abstract

Wearable tear-based biosensors have garnered substantial interest for real time monitoring with an emphasis on personalized health care. These biosensors utilize major tear biomarkers such as proteins, lipids, metabolites, and electrolytes for the detection and recording of stable biological signals in a non-invasive manner. The present comprehensive review delves deep into the tear composition along with potential biomarkers that can identify, monitor, and predict certain ocular diseases such as dry eye disease, conjunctivitis, eye-related infections, as well as diabetes mellitus. Recent technologies in tear-based wearable point-of-care medical devices, specifically the state-of-the-art and prospects of glucose, pH, lactate, protein, lipid, and electrolyte sensing from tear are discussed. Finally, the review addresses the existing challenges associated with the widespread application of tear-based sensors, which will pave the way for advanced scientific research and development of such non-invasive health monitoring devices.

## 1. Introduction

**Figure 1:**
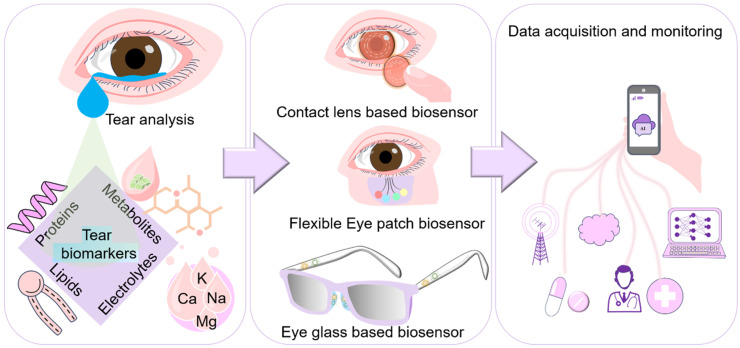
Schematic illustration of tear-based biosensors showing contact lens, flexible eye patch, and eye glass-based biosensors coupled with the real time data acquisition through a smartphone camera.

With the tremendous development in the field of flexible electronics, wearable biosensors are progressing towards prospective technologies in the field of non-invasive health monitoring [1,2,3,4,5]. Wearable biosensors can continuously track real-time physiological information via dynamic as well as non-invasive measurements of various biochemical markers in biofluids [6,7]. In recent years, there have been rapid developments of wearable electronic devices that can accurately monitor vital physical (heart rate, body temperature, blood pressure, electrical signals, intraocular pressure) and chemical signals (from saliva, blood, tear, sweat, urine) and convert into an electrical signal to understand human health conditions [8,9,10,11,12]. Biofluids or biological body fluids (such as sweat, tears, saliva, interstitial fluids) secreted by organisms, composed of various biochemical components, are of interest to understand the body’s deeper biomolecular state. Among these, tear-based ocular wearable biosensors are an emerging non-invasive technology focusing on analyzing tear composition and secretion to provide valuable health insights, as tears are a prospective candidate for wearable device applications [13,14,15,16,17]. In addition to its beneficial aspects in being used for the pre-diagnosis of ophthalmological disorders (dry-eyes, glaucoma, rosacea, aniridia, and keratoconus), tear fluids can be utilized to identify oncological (breast, prostate, lung, ovarian, and colon cancers), neurological diseases (multiple sclerosis, Alzheimer’s, and Parkinson’s disease), and renal dysfunction [18,19,20,21]. Furthermore, other pertinent details associated with various clinical implications apart from the scope of disease detection can be obtained using tear biomarkers such as monitoring disease progression, information associated with treatment decision, and prediction of clinical outcomes. For instance, reduction in protein (lysozyme and lactoferrin) levels in tears can correlate with elevated infection rates in dry-eye disease (DED) patients, pointing towards further investigation of microbial attack [22]. With regard to the progression of disease, early DED tears are characterized by depleted content of whole protein, lipid binding proteins, and some inflammatory associated proteins [23]. In addition, tear biomarkers can be used to predict clinical outcomes, for instance FDA-approved InflammaDry, which is already in clinical use and can help make decisions on anti-inflammatory therapies by providing details on the inflammatory status of the eye [24]. Apart from collecting and examining samples using conventional diagnostic methods in a hospital, tear-based ocular wearable biosensors detect the biomarkers present in tear fluid, thereby allowing patients to diagnose their disease at home and monitoring its intensity for a required therapy [15,25]. Such devices, termed as point-of-care devices, provide results within a short time and are exempted from the requirements of experts or laboratory staff [16,26]. The majority of the wearable biosensing point-of-care systems are contact lens-based sensors used to assay various tear biomarkers [27,28]. Such systems, having direct contact with the ocular surface, not only measure the biomarkers (proteins, lipids, metabolites, and electrolytes) but also detect vital signs (blood pressure, pulse, and temperature) and biological signals (electroretinogram and electrooculogram) [29,30]. These devices incorporate all the necessary components for the purpose of biosensing, data processing, and power source management for the detection of analytes to achieve the desired wearable system (Figure 1).

The scope of this review focusses on non-invasive wearable tear-based biosensors pertinent for point-of-care health applications. The review discusses and provides insights into the various aspects of tear fluid, constituent biomarkers, and prospective sensing technologies based on these biomarkers. Despite several published reviews covering the broader aspects of wearable biosensors [1,6,31,32,33,34,35], there has been a limited number of review articles [14,36,37] exploring the various facets of tear-based biosensor. Since the present review is focused on the use of tear-based sensors to monitor chemical signals from various biomarkers, research works related to intraocular pressure monitoring are not covered herein. With this review, we hope to provide researchers in this field a clear understanding of the basic concepts along with recent developments and challenges to be addressed, which can be translated to potential innovative point-of-care technologies.

## 2. Insights into Tear

Tear (also termed as tear fluid or tear film) is the outermost thin biofluid layer covering the epithelial cells. Tear is a complex body fluid containing abundant biomarkers that are closely associated with ocular and systemic health condition. Tear film covering the ocular surface is vital in (a) protecting the eyes, (b) lubricating eyelids, conjunctiva, and cornea (thereby preventing drying of ocular surface epithelia), (c) preserving a smooth surface to refract the light rays, and (d) maintaining conjunctiva and avascular cornea in a healthy condition by supplying nutrients [38]. This film acts as a protective shield on the ocular surface, protecting against foreign substances, providing excellent antifouling properties, and it exhibits remarkable microbial resistance. Microbial-resistant factors such as IgA, IgG, IgE, lysozyme, lactoferrin, transferrin, ceruloplasmin, glycoprotein, and anti-proteinase are present in the tear film aqueous layer [39]. Secretion of tear occurs at a rate of 1–2 μL/min, having a thickness of about 3 μm and volume of 3–10 μL [40]. Tear fluid is predominantly generated by plasma cells of the immune system, lacrimal glands, and conjunctival cup cells [41]. This is followed by tear evaporation or draining via the lacrimal puncta. Compared to blood composition, tear is relatively less complex, rendering it as a promising candidate for non-invasive theranostics [39]. There are three different types of tears: basal tears, reflex tears, and closed eye tears. Basal tears on the ocular surface (a) supply nutrients, (b) maintain ocular relief, and (c) remove debris, whereas reflex tears are secreted in response to irritants such as foreign bodies or chemicals and are responsible for flushing irritants from the ocular surface [42,43]. Closed eye tears lubricate eyes during sleep. Prolonged lid closure during sleep lowers tear pH (normal pH value ~7.45) due to carbon dioxide accumulation [44]. Tear film components (lysozyme, lipocalin-1, and lactoferrin) and osmolarities are more or less similar among these three types of tears. However, the number of proteins, lipids, and secretory IgA varies, with proteins and lipids being higher in basal tears [45,46].

**Figure 2:**
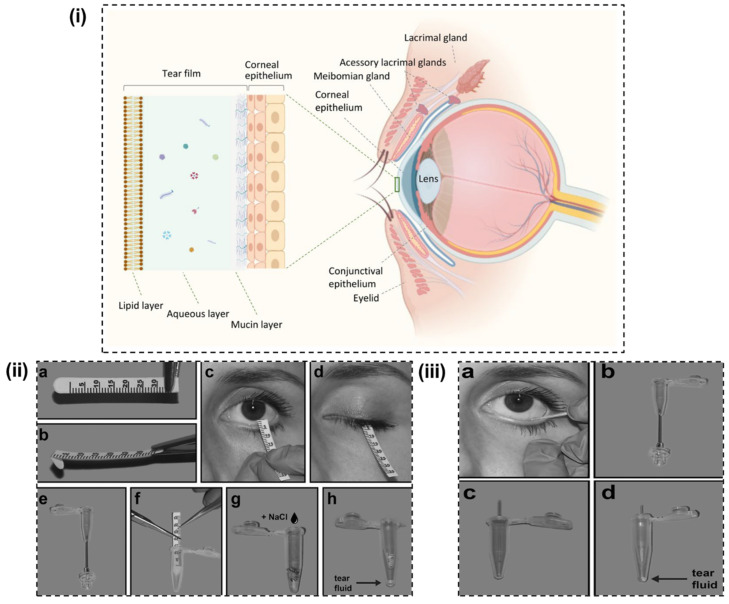
(**i**) Structure of three layered tear film constituting of inside mucin, intermediate aqueous, and exterior lipid layer (Reprinted with permission from [52]), tear fluid collection steps using (**ii**) Schirmer’s test strip and (**iii**) microcapillary tube methods (Reprinted with permission from [53]).

Tear film is heterogeneous and possesses a layered structure (Figure 2i) comprised of an inside mucin layer, an intermediate aqueous layer, and an exterior lipid layer [47]. Mucins form the inner layer, which are secreted largely by goblet cells present in the conjunctival epithelium. In addition, acinar cells present in the lacrimal gland and epithelial cells of cornea and conjunctiva also contribute to a smaller extent. The mucin layer stabilizes and affixes the middle aqueous layer to the corneal epithelium via glycocalyx to facilitate consistent lubrication. This layer, comprised of glucose, urea, salts, immunoglobulins, and proteins, increases the overall stability and lowers surface tension [48]. The middle aqueous layer lubricates and protects the ocular surface, which is composed of glucose, inorganic salts, metabolites, proteins, oxygen, and electrolytes such as magnesium, bicarbonate, calcium, and urea [40,49]. Tear film mostly exists in the aqueous phase, comprising a distinct mucin concentration that depends on this layer and the thin superficial lipid layer. The outer superficial lipid layer, with a thickness of 50–100 nm [50], is the environmental-tear interface. Here, lipids are mostly produced by meibomian glands, which serve a major role in maintaining homeostasis [51]. Meibomian gland secretions or meibum constitutes both polar as well as non-polar lipids such as phospholipids, triacylglycerol, wax esters, free fatty acids, diesters, and cholesterol. Meibum, which spreads onto the tear film, reduces the tear evaporation rate and also smoothens the corneal surface, forming a protective eye barrier from dust, pollens, and microbial agents.

## 3. Biomarkers in Tear

Though comparatively less complex than blood, tear still contains a significant level of biomarkers, which encompass proteins, lipids, electrolytes, and metabolites as a result of both secretion and passive leakage from the blood [54,55]. These biomarkers reflect various ocular diseases, which possess significant clinical importance to human health. Detection and diagnosis of biomarkers in tear are dependent on sampling techniques. Schirmer’s test strip and microcapillary tube methods (Figure 2ii,iii) are commonly used for tear sample collection [53,56]. Schirmer test strip consists of a paper test strip that is placed in the eye to measure the amount of tear production, whereas the latter method makes use of thin narrow tubes that are inserted gently into the lower eyelid to collect tear via capillary action. Though the microcapillary tube method is considered to be more comfortable and less invasive compared to Schirmer’s strip method, Schirmer’s strips are still the commonly implemented method due to easy handling during sample collection, facile execution during the clinical routine, and that they can additionally evaluate tear fluid volume as well as provide information regarding the dry eye status of patients [57,58]. Each sampling method has its own advantages and limitations; hence it is imperative to select the suitable tear collection method in accordance with the suitability for a specific problem.

### 3.1. Proteins

Study of protein biomarkers in tear fluid is one of the major focus areas in the clinical proteomics as it is more related to several distinctive ocular disorders such as DED, glaucoma, Sjogren’s syndrome, and other systemic diseases such as diabetes and cystic fibrosis–related diabetes [30,59,60,61]. Tear proteins are present in the range of 6–11 mg/mL [59], with up to about 1543 types being detected [62]. Depending on the nature of ocular disease, analyte quantity, specific protein biomarker, and measuring technique, sensitivity (true positive rate) and specificity (true negative rate) of tear biomarkers vary. For instance, protein biomarkers exhibited a varying sensitivity (50–90%) and specificity (70–90%) for identifying DED [63]. The key protein biomarkers in tear include lysozyme (the most predominant biomarker, constituting about 25% of total proteins), along with lactoferrin, lipocalin, secretory immunoglobulin A (sIgA), and cystatin [64,65]. Lysozyme is a ubiquitous enzyme with excellent antimicrobial properties, which shields the eye against bacterial diseases. The reduction of lysozyme in tear can lead to DED, and is often used as an indicator of tear quality and eye health [66]. Changes in lysozyme level indicate the variation in tear composition or response to bacterial infections and inflammations. The molecular weight of lysozyme ranges from 14–15 kDa and it is a globular protein with primary sequence consisting of 129 amino acids with a net positive charge under neutral pH (isoelectric point of 11) [67]. Time-lapse imaging using an economical and portable reader is reported to be used to quantify lysozymes, which are non-specifically bound to the contact lens [68]. Ballard et al. [68] reported the quantification of lysozyme in human tear-fluid with the aid of contact lenses using a field-portable reader. A lower value of lysozyme concentration of 2.43 ± 1.66 μg/mL was obtained in a patient affected with DED compared to a healthy adult (6.89 ± 2.02 μg/mL).

Lipocalin present in tear is a lipid-binding protein with lower tear surface tension, enabling it to protect the underlying epithelium. This lipocalin-lipid bond favors lipid solubility, thereby establishing a rapid equilibrium during the blinking process and improving the development of a homogenous, compact outer lipid layer, which monitors tear evaporation rate [66]. This low-molecular weight protein (18–40 kDa) has a large ligand binding cavity or calyx, which can bind to other tear protein macromolecules such as lysosome and lactoferrin [69]. Lipocalin is the principal phosphoprotein present in tear fluid, which is one of the biomarkers present in the tears of diabetic retinopathy patients [70]. Lactoferrin is an iron-binding protein (82 kDa) that exhibits antioxidant activity, is microbial-resistant, and has anti-cancer and anti-inflammatory properties, allowing it to play a major role in maintaining ocular health [71]. With an average concentration of about 1.42 mg/mL in a healthy individual, lactoferrin maintains tear film stability and its lower levels are related to DED and other ocular surface disorders [72,73]. Being an Fe-binding protein, lactoferrin displays scavenging activity against oxygen free radicals and hydroxyl, thereby improving the antioxidant mechanism and allowing it to retain tear homeostasis, making it helpful when combating DED [72]. Lactoferrin, with a threshold level of 1.1 mg/mL, displays a high sensitivity (79.4%) and specificity (78.3%) for DED diagnostics [74]. Similarly, lactoferrin in conjugation with Schirmer’s test yields increased sensitivity (72%) and specificity (95%) for Sjögren’s syndrome [75]. Lebrecht et al. reported the usage of 20 different protein tear biomarkers for early detection of breast cancer with high specificity and sensitivity (approx. 70%) [76]. Fluorescence-based biosensing is one of the methods commonly employed for detecting lactoferrin in tears [77]. Shi et al. [78] employed a contact lens-based fluorescence lactoferrin biosensor for tear fluid collection and analysis. Lactoferrin in tear has been detected in the range of 0 to 5 mg/mL, with a strong linear relation, and can be obtained with a limit of detection (LOD) of 0.44 mg/mL. Secretory immunoglobulin A (sIgA) is an antibody that is crucial for mucosal immunity, which helps to prevent infections by binding to pathogens. Variations in sIgA levels indicate inflammatory or infectious conditions affecting the ocular surface. Cystatin is a proteinase inhibitor that helps to regulate proteolytic enzymes and has roles in inflammation and tissue repair, an increased concentration of which, in tears and intraocular fluids, is reported for patients with choroidal melanoma [79]. These protein biomarkers serve as crucial indicators of eye health and systemic conditions. Albumin is a major plasma protein involved in maintaining osmotic pressure and transporting various substances. The elevated levels of albumin in tears can indicate leakage from blood vessels or inflammation. In addition, variation of cytokines or chemokines in tear composition acts as potential biomarkers to detect various ocular diseases [80,81]. Cytokines in tears are used for understanding and diagnosing various ocular conditions, including DED, allergic conjunctivitis, infections, and autoimmune disorders [82,83,84,85]. By analyzing cytokine levels, clinicians can gain insights into the inflammatory and immune processes affecting the eye and develop treatment strategies accordingly. Interleukins (ILs) (Interleukin-1 (IL-1), Interleukin-6 (IL-6) and Interleukin-10 (IL-10)), Tumor Necrosis Factor-alpha (TNF-α), and Transforming Growth Factor-beta (TGF-β) are the major key cytokines present in tears, which are responsible for regulating immune as well as inflammatory activities [86,87]. IL-1 is a dominant pro-inflammatory cytokine that activates other immune cells and generates additional inflammatory mediators. IL-6 is a multifunctional cytokine that promotes inflammation and helps in tissue repair and regeneration, thereby affecting various immune responses and inducing acute phase response. IL-10 is another anti-inflammatory cytokine that modulates and suppresses excessive immune responses, helping in limiting inflammation and promoting tissue repair. TNF-α is a key pro-inflammatory cytokine for various inflammatory and autoimmune conditions, whereas TGF-β is associated with regulation of cell growth, differentiation, and immune responses. Chemokines are signaling molecules with major key factors such as C-C Motif Chemokine Ligand 2 (CCL2), C-X-C Motif Chemokine Ligand 8 (CXCL8), C-X-C Motif Chemokine Ligand 10 (CXCL10), and C-C Motif Chemokine Ligand 5 (CCL5) [88,89]. Gabriela et al. [90] reported that lower levels of CXCL10 were associated with worse ocular symptoms, whereas reduced CCL2 can be correlated with positive ocular target tests in patients affected by Sjögren’s syndrome. Despite this, the uncertainty and unknown characteristic of proteomics in tears delimit the application of protein biomarkers for clinical applications.

### 3.2. Lipids

Lipids are pivotal in maintaining a stable tear film by forming an outer layer, known as the tear film lipid layer, that helps reduce surface tension, facilitating its respreading in between blinks and preventing evaporation [50]. Tear film lipid layer (average thickness of 42 nm) acts as an evaporation-resistant barrier (inhibiting evaporation by about 4–20 fold), preventing eyes from drying, and assisting in stabilizing the tear film. Lipids are composed of triacylglycerols (TAGs), phospholipids, and cholesterol, which help in favoring cellular energy provision and lipid metabolism [91,92]. TAGs represent the primary form of lipids in the lipid layer and help prevent the aqueous layer beneath it from being evaporated, thereby maintaining proper hydration of the ocular surface. Phospholipids contain lecithin, which helps in reducing the tear film surface tension. Cholesterol maintains the stability and fluidity of the lipid layer. Changes in this lipid composition can be a biomarker for DED, blepharitis, or meibomian gland dysfunction [93]. Cholesterol monitoring can be conducted with the help of tear fluid. Cholesterol in tears exists as cholesteryl ester and free cholesterol (7:3 ratio). Overall cholesterol levels in tear can be ambiguous (in comparison with blood) due to this considerable amount of cholesteryl esters in tear, hence a discerning detection of free cholesterol in tears is necessary. There are limitations associated in discerning the quantitative information of compositional variations between lipids generated by Meibomian gland and lipids present in tear fluid, largely associated with person-to-person compositional variation, paucity of sample, and complexity of lipid species.

### 3.3. Electrolytes

The major electrolytes found in tear include chlorides and bicarbonates of Na^+^ (120–170 mM), K^+^ (6–42 mM), Ca^2+^ (0.3–2 mM), and Mg^2+^ (0.3–1.1 mM). An increased concentration of these electrolytes and osmolarity can elicit DED [94]. Monitoring these in tears can offer insights into hydration status, electrolyte imbalances, and overall health. The presence and proper balance of electrolytes in tear fluid are essential for proper ocular function. Elevated tear osmolarity and tear film instability are primarily linked with higher levels of monovalent electrolytes such as NaCl or KCl, which can disrupt large molecule to electrolyte ratios in tear [95]. Several divalent cations are reported to develop a non-covalent interaction with charged anionic surface molecules such as mucin, thereby altering the structure and function of such molecules [96]. Harvey et al. [97] utilized fiber optic sensing technology for tear electrolyte analysis using ex vivo contact lenses as a sample source to quantify potassium in tears. Potassium detection was based on displacing sodium from a water-soluble sodium complex along with tetraphenylborate anion to develop insoluble potassium tetraphenyl borate, which, when dispersed in water, scatters light. The fiber optic technique can quantify signal change relative to precipitate content. Such technologies can be expanded as potential platforms for detecting other tear electrolytes, for instance calcium sensors, possibly linked with magnesium. More research in the field of tear electrolytes is highly desirable for developing promising point-of-care non-invasive wearable systems to detect and monitor various ocular diseases.

### 3.4. Metabolites

Metabolites as tear biomarkers are used in many non-invasive biosensors for sensing glucose and lactate [98,99,100]. Glucose is an extensively used biomarker for diagnosing diabetes metabolic disorder, whereas lactate is used for the analysis of the health conditions of athletes. Lane et al. reported the standard glucose concentration of 0.16 ± 0.03 mmol/L and 0.35 ± 0.04 mmol/L in tears for non-diabetic and diabetic patients, respectively, and found that the values differ according to the method of collection of tears and the measurement technique [98]. Chen et al. reported the presence of about 60 metabolites in human tear fluid, including but not limited to amino acids, peptide, phospholipids, L-lactate, purines, and quaternary amines [101]. Metabolomics is a promising research direction that can yield useful details pertaining to DED, meibomian gland disfunction, kerataconus, and ocular surface diseases [102,103]. Karamichos et al. first identified the role of variations in endogenous metabolites, which are oxidative stress indicators, such as glutathione in tears of keratoconus patients [104]. Ma et al. [105] reported the promising role of tear metabolites such as phosphatidylcholine, sphingomyelin, lactosylceramide, and docosahexaenoic acid as potential biomarkers to detect metabolic dysregulation associated with ocular chronic graft-versus-host disease. Lactate is one other tear metabolite capable of indicating oxygen deficiency and lactic acidosis due to lactate accumulation [106]. Tear lactate should be in the range of 1–5 mM, which is relatively large when compared to lactate in blood [44,100]. Disparity in lactate levels relate to many ocular diseases; for instance, a reduced value indicates de-epithelialized cornea [107]. Due to intensive exercise or physical strain, lactate variations occur that point towards oxygen deficiency or high salt concentration, reflecting the metabolic state of human body. A detailed description of various works in the field of tear-based glucose, pH, lactate, protein, lipid, and electrolyte sensing is provided in Section 4.

## 4. Tear-Based Wearable Bio-Sensing Technologies

Wearable tear-based biosensors provide a non-invasive platform to measure biomarkers or analytes present in the tear fluid, and are useful for monitoring physiological condition and tracking real-time health conditions [6,108]. Sensing systems for such biosensors are commonly fabricated using conventional standard microelectromechanical system (MEMS) technologies, which include various sub-sections including vacuum deposition, photolithography, and wet/dry etching [109]. The integration of sensing elements into flexible platforms for wearable sensors can be achieved via printing, sputtering, and photolithography. The widely used microfluidic platforms for tear-based wearable sensors can be fabricated based on soft lithography, micro-milling, 3D printing, laser micromachining, and injection molding [110]. Electrochemical, optical, or fluorescence-based detection techniques have been widely researched to analyze tear fluid [77,111]. These sensors are precisely small and are often integrated into contact lenses or adhesive patches (which adhere to the skin beneath the eye). Contact lens-based biosensors are non-invasive and provide a direct sample interface for a continuous monitoring and allow for the integration of various sensing modes. Contact lenses should be well fitted onto the eye surface, where it rests on a continuous aqueous tear layer covered by the lens and epithelium. It should be coated with an external superficial lipid layer to protect the aqueous tear layer. Contact lens sensor depends on the accurate collection of tear and its constituents to directly measure various parameters such as glucose concentration, pH, proteins, ions, intraocular pressure, and corneal temperature [112]. Owing to itscomfortability, reliable tear yield, better oxygen permeability, and potential to bestow precise continuous monitoring, contact lens-based systems emerge as a potential platform to develop tear-based sensing technologies [113,114]. In addition to contact lens sensors, a non-invasive eyeglass-based wearable sensor was employed for real-time monitoring of different target analytes in tears [14,115]. A wearable tear biosensor incorporated with a microfluidic electrochemical-based detector incorporated onto an eyeglass nose-bridge pad is developed by Wang et al. to monitor vital biomarkers in tear [14]. The device system consists of a sensor system that is mounted on eyeglasses, converting ethanol from alcohol (orally administered) present in tear fluid into acetaldehyde and H_2_O_2_ with the help of alcohol-oxidase enzyme. Upon filling the device reservoir with tear fluid, the integrated biosensor with immobilized alcohol-oxidase senses the level of alcohol. This study aims to monitor tear analytes outside the eye region, thereby overcoming the limitations such as high risk of infection and vision impairment associated with contact lens systems. In addition to these, strip-based ocular sensors based on hard plastic substrates have been used for monitoring glucose, keratoconjunctivitis sicca, and transcutaneous oxygen in tears [116]. However, strip-based sensors lack data processing capabilities and cause eye irritation due to the substrate, and hence are not a viable option to be used as a wearable device [36]. The subsequent sections of this review elaborate various tear-based ocular wearable sensors.

### 4.1. Glucose Monitoring

**Figure 3:**
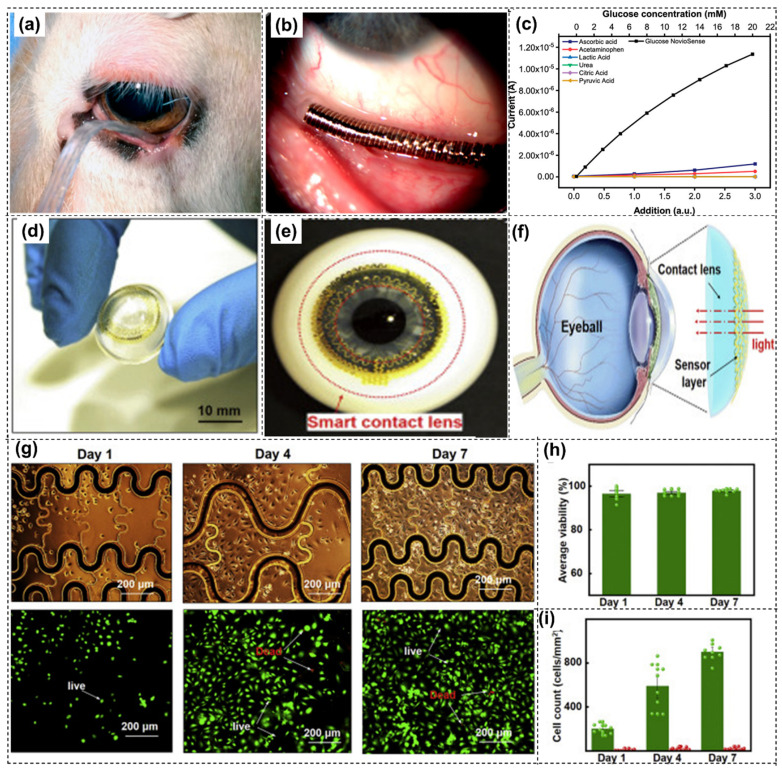
(**a**) Preclinical evaluation of NovioSense tear glucose sensor in sheep, (**b**) clinical trial–phase II evaluation in human eye using tear glucose sensor, (**c**) NovioSense device relative response to physiological interferences and glucose at varying concentration (reprinted with permission from [119]), (**d**) digital image of the sensor layer transferred onto a dome-shaped PDMS substrate, (**e**) image of the sensor placed on an artificial eye, (**f**) schematic representation of optical transmittance testing, (**g**) optical images (upper) and fluorescent images (lower) of in vitro cytotoxicity evaluation of contact lenses using human umbilical vein endothelial cells at different days (green and red fluorescence shows live and dead cells respectively), (**h**) average cell viability (percentage of live cells remain similar for 7 days), and (**i**) number of cells present (increasing number of live cells indicates cell culture reliability) (reprinted with permission from [125].

Diabetes mellitus, a chronic metabolic syndrome caused by genetic factors, lifestyle habits, and immune dysfunction, is a condition of global concern that impacts approximately 537 million people [117]. It is a major health disorder that can even lead to irreversible vision loss, and hence regular monitoring of tear glucose concentration avoids such adverse health conditions. Non-invasive tear-based glucose biosensing has recently emerged as a mode of interest for developing wearable electronics for continuous glucose monitoring of diabetes patients [118]. This sensing method is an alternative to traditional self-monitoring of blood glucose levels. The NovioSense Glucose Sensor is the very first sensor reported for continuous measuring of glucose levels in the basal tear fluid and it correlates glucose in blood with clinical viability [119]. The sensor uses a traditional enzymatic detection of glucose, which consists of a miniature spring-like electrochemical sensor constituting several coiled wire electrodes shielded using a protective polysaccharide-based hydrogel. The polysaccharide-based coating additionally constitutes immobilized glucose oxidase as the enzymatic sensing element. Glucose oxidase converts glucose and oxygen into gluconolactone and hydrogen peroxide, and hydrogen peroxide is subsequently oxidized. The electrode then utilizes the dissociated ions for quantifying the glucose concentration using a chronoamperometric measurement. This sensor can be conformally placed beneath the lower eye lid to continuously access tear, which has exhibited promising clinical viability in animals and humans [Figure 3a,b]. The sensor is coupled with wireless data transmission for measuring tear glucose levels. The sensor exhibited glucose measurements in sub mmol concentrations relative to blood glucose levels and high tolerance to interferences (Figure 3c). Similar to the NovioSense glucose sensor, Google and Novartis [120] developed smart contact lenses to measure tear glucose levels for diabetic patients. This sensor system consists of a lens fabricated using a hydrogel-based small wireless chip, a miniaturized glucose sensor, and a small battery sandwiched between lens periphery layers. Tear seeps out through the pin-hole present in the lens into the sensor, which generates glucose readings and transmits them to a smartphone. However, this study lacks the capacity to provide reliable readings and a clear correlation of glucose in blood and tear fluid.

Continuous glucose monitoring in tear biofluid is predominantly achieved using optical as well as electrochemical detection techniques. Glucose levels in tear can be used to identify the diabetic stage of patients (normal range ~0.1–0.6 mM for a healthy adult and >0.6 mM for a diabetic patient), allowing for suitable therapy by suitable treatment decisions [121]. Similar to sweat glucose, tear sensors can quantify tear glucose levels and correlate with blood glucose levels using similar correlation coefficients based on artificial collection techniques, physico-chemical stimulations, and naturally occurring accumulations [114]. Contact lenses are commonly used for tear glucose detection to analyze and monitor glycemic control. As the contact lens always has direct contact with the tears and does not induce discomfort to eyes, contact lens-based glucose sensing devices are more preferred for tear collection [113]. Contact lenses embedded with fluorescent nanoparticles have been explored to determine glucose concentration for ocular and systemic physiological detection [77,122]. The first developed fluorescent glucose biosensor was a contact lens embedded with boronic acid, consisting of fluorophores to measure the tear glucose concentration [123]. Ruan et al. [124] developed a novel glucose sensing contact lens system of crystalline colloidal array embedded within a hydrogel matrix attached to a rigid gas permeable contact lens. The system exploits a linear correlation between glucose concentration and diffraction wavelength to shift between 567–468 nm with varying glucose content. Glucose-detective gelated lenses selectively detect visible color changes between reddish yellow, green, and blue with variation in glucose concentration between 0 and 50 mM. Guo et al. reported an ultrathin MoS_2_ transistor incorporated within a contact lens with a glucose sensing unit using GOx as the bio-enzyme [125]. PDMS substrate was used for the sensor layer to be transferred to obtain a dome shape with dual enclosed stainless-steel hollow hemispheres (Figure 3d,e). This sensor system included a photodetector for receiving optical data (Figure 3f), a glucose sensor to monitor glucose levels from tear, and a temperature sensor to check for corneal infection. The sensor displayed significant current changes, pointing towards high sensitivity and high stability with varying glucose concentrations. The contact lens exhibited 93% optical transmittance with better biocompatibility when tested with human umbilical vein endothelial cells (Figure 3g–i).

Similarly, electrochemical sensors integrated with hydrogel-based contact lenses yield promising health care data [126]. Integrating such electrochemical sensors into contact lens-based technologies along with electronic components can measure basal tears directly. Electrochemical biosensors using amperometric, potentiometric, voltametric, photoelectrochemical, and electro-chemiluminescent-based methods are being used to detect chemical biomarkers in tears [126,127]. Owing to their speedy response, accuracy, improved sensitivity, and ability to be miniaturized, electrochemical sensors remain as a core research arena for developing glucose monitoring devices [128]. Due to their selectivity, sensitivity, and rapid response, electrochemical sensors utilize specific selective enzymes such as glucose oxidase (GO_x_) and glucose dehydrogenase for selective glucose detection. During the enzymatic reaction, glucose is converted into gluconolactone and hydrogen peroxide, where hydrogen peroxide dissociates to produce hydrogen ions, oxygen, and electrons. The three-electrode system utilizes the dissociated electrons for quantifying the glucose concentration. Kudo et al. [129] developed a flexible and biocompatible glucose biosensor using functional polymers such as polydimethyl siloxane, 2-methacryloyloxyethyl phosphorylcholine, and dodecyl methacrylate. The sensor, which was fabricated by the immobilization of GO_x_ on the polymer matrix, correlated output current to glucose level over a range of 0.06 to 2.00 mM/L (correlation coefficient of 0.997), which covers the normal tear fluid glucose concentration. In another study, a glucose-responsive hydrogel electrode was developed by Kajisa et al. [130] that displayed high sensitivity and biocompatibility. The optimized electrode showed glucose responsivity with high sensitivity of 7 mV per decade of glucose concentration and suppressed the nonspecific adsorption of albumin, proving that the electrode can be applied in wearable devices to detect glucose in tears. Mitsubayashi et al. [131] fabricated a glucose sensor by immobilizing GO_x_ with glutaraldehyde solution onto a transparent thin film oxygen electrode to detect tear glucose. The sensor, with a flexible structure and good optical transparency, measured glucose range of 0.06 to 1.24 mM covering the normal tear glucose level (0.14 mM) with good reproducibility. A non-enzymatic biosensor, utilizing nanomaterials as nanozymes, aids in enhancing the electrochemical properties of non-invasive tear-based glucose sensors compared to enzyme-based biosensor. Highly sensitive electrochemical tear-based glucose biosensors based on transition metal oxides, noble metals, conductive polymers, and biomass-derived carbon are being researched for continuous glucose monitoring [27,132,133,134,135]. Noble metal-based biosensors possess improved electrocatalytic activity and higher sensitivity, selectivity, and linear range of detection. Among the noble metals, Pt and Au exhibit better sensing performance and accuracy in tear glucose sensors [136,137]. Metals are being used successfully for doping and loading with polymers for improving sensing performance [138]. For instance, Au@Pt nanoparticles were loaded into hyaluronic acid hydrogels to fabricate smart contact lenses for continuous glucose monitoring [135]. In this study, glucose variations in the tear of diabetic rabbits were evaluated to be in the linear range of 0.01–50 mg/dL. This lens also proved clinical feasibility and yielded 94.9% acceptability in wearing on eyes of diabetic patients. As these metals are precious and expensive, fabricating such novel material composition for improving the efficiency remains a challenge. Transition metals such as Fe, Co, Ni, Cu, and their oxides are promising materials due to their cost-effectiveness, improved electrical conductivity, high selectivity, enhanced catalytic activity, and facile fabrication [139,140]. Transition-metal based contact lenses using γ-Fe_2_O_3_@NiO magnetic oxide nanosheets as sensing material were fabricated and tested in vivo for non-enzymatic tear-based glucose monitoring [141]. The sensor was capable of linearly detecting glucose with a reliable glucose detection limit of 0.43 μmol, confirming the potential for innovative nanosystems to be used for tear glucose monitoring. Carbon nanomaterials-based sensors are also promising candidates in this research direction, owing to their improved physico-chemical, electrical, and mechanical characteristics [142,143]. Despite their inherent properties, carbon nanomaterials are still associated with toxicity concerns, which can potentially affect the biocompatibility aspects. Hence, biocompatible materials along with meagre biofouling properties need to be developed for promising tear-based glucose sensors.

### 4.2. pH Level Monitoring

**Figure 4:**
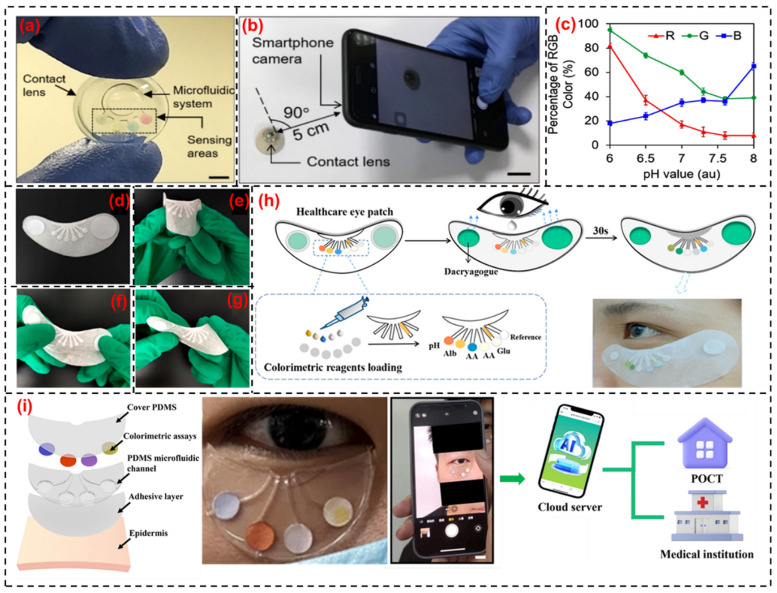
(**a**) Photograph of the microfluidic contact lens with colorimetric sensor (scale bar—5 mm), (**b**) smartphone camera imaging the color variation of sensor (scale bar—1 cm), (**c**) colorimetric characterization of pH sensor ranging from dark yellow at pH 6 to blue at pH 8 (reprinted with permission from [147]), (**d**–**g**) mechanically flexible eye patch sensor depicting tensile, torsional, and bending nature, (**h**) assay process to trigger the chromogenic reaction using the flowing tear stimulated by dacryagogue followed by the removal of eye patch sensor after 30 s for data collection and analysis (reprinted with permission from [151]), and (**i**) multilayered structure of crescent shaped PDMS microfluidic colorimetric sensing patch attached under the right eye of the human face followed by color data acquisition with the aid of a smartphone camera assisted by deep learning artificial intelligence (reprinted with permission from [17]).

Measuring pH level in tears can assist in monitoring conditions related to eye health or other systemic conditions (diabetic retinopathy) and is also important in determining ocular penetration of drugs. Healthy tear pH values range from 6.5–7.6. Tear pH sensors display green color for healthy eyes, yellow color in a slightly acidic pH, and blue color in an alkaline pH [144,145]. A decrease in pH may reveal corneal infection, dry eye syndrome, or ocular surface inflammation [146]. Alkaline tear pH is a potential indicator for pre-diagnosis of rosacea, which is a chronic dermatosis condition, and epidemic or herpetic keratoconjunctivitis [147]. Healthy individuals reported a tear pH of 7.0 ± 0.8; those individuals with non-treated rosacea report a tear pH of 8.0 ± 0.32 [145]. Tear pH can be evaluated with the aid of (a) pH-sensitive fluorescent probes combined with a fluorometer, (b) facile colorimetric pH monitoring poly-HEMA contact lenses cross-linked with an anthocyanin dye, and (c) electrochemically, using a micro pH meter [126]. Riaz et al. [148] reported the development of soft contact lenses based on poly-2-hydroxy ethylmethacrylate functionalized with anthocyanin pigments to monitor ocular pH, and the lenses displayed color shift in response to pH variations of 6.5, 7.0, and 7.5. Semi-quantitative microfluidic contact lens sensors were reported [147] to detect pH based on methyl red (MR), bromothymol blue (BB), and phenolphthalein (PP). MR, which is comprised of carboxylic acid and amine groups, serves as an acid or base to detect pH (4.3–6.2). BB forms triphenylmethane in an alkaline environment and changes the color from yellow to blue. PP induces a color change from a colorless lactonic ring structure to quinoid-carboxylated structure with an increase in pH. A sensor comprised of a microfluidic system integrated with a contact-lens and smartphone-based data acquisition system (Figure 4a,b) reported a sensitivity of 12.23 nm/pH and LOD of 0.25 pH units. The sensor was tested for a pH range of 6–8 and the corresponding colorimetric characterization is shown in Figure 4c. Flexible, self-healing potentiometric sensors in the form of cables were weaved from carbon fiber thread electrodes coated with self-healable polymers composed of poly(1,4-cyclohexanedimethanol succinate-co-citrate) [149]. Sakr et al. developed a vat photo polymerization-based 3D printing based on neutral red dye-doped hydrogel, which displayed an optimal pH response and mechanical stability using 0.45 mm 3D printed disks [150]. The underlying phenomenon responsible for the pH responsive behavior of neutral red dye is based on chromophore protonation/deprotonation. With pH change, dye molecules can accept or lose protons, thereby effectuating an electronic structure alteration and an ensuing color change (chromophore protonation / deprotonation).

One limitation associated with the colorimetric tear sensor is the change in color of chromogenic agents on reaction with target biomolecules, giving rise to variation in colors even with same concentration of target biomarkers. A flexible eye patch biosensor (Figure 4d–h), which comfortably fit in the skin below the eyes, was reported by Xu et al. [151]. This biosensor displayed pH monitoring (based on hydrogen ion sensing) in the range of 5.4–8.0 (orange red to green). pH indicators such as methyl red (MR), bromothymol blue (BTB), and phenolphthalein (PP) were evenly mixed with cetyltrimethylammonium bromide (CTAB), which acted as a fixative to block the diffusion of chromogenic agents into the sensing region to address the problem mentioned above. Quantitative measurements with an improved sensitivity are reported by capturing the pH-associated color signal change using a smartphone and analyzing it in the sensing region, rendering a high precision compared to pH paper strip indicators. In another recent work by Wang et al., artificial intelligence assisted wearable microfluidic colorimetric sensor technology to monitor several pivotal tear biomarkers, in which pH is also included [17]. A PDMS flexible microfluidic colorimetric sensing patch composed of a mixture of three acid-base indicators (MR, BTB, and PP) as the chromogenic reagents was coupled with a cloud server data analysis system on a smartphone for color data analysis (Figure 4i). Despite tear pH offering a promising analyte for non-invasive monitoring of physiological parameters, there are limitations associated with collecting sufficient tear fluid for efficient pH measurement due to lower concentrations of H_3_O^+^ present [152].

### 4.3. Lactate Monitoring

Monitoring lactate levels can be useful for athletes and individuals to track their metabolic status and hydration levels. As mentioned in Section 3.4, lactate monitoring is of great importance to evaluate hypoxia conditions such as cancer, bacterial/fungal infection, cerebral stroke, and trauma [153,154,155]. Though lactate levels are often analyzed using blood, tear fluid is more compatible and accessible. Tear fluid removes l-Lactate from the body through diffusion across the stroma and endothelium. Lactate detection commonly uses an enzyme named lactate oxidase (LO_x_) in electrochemical biosensors, in which LO_x_ is typically immobilized onto an electrode surface [156]. This enzyme specifically catalyzes lactate-to-pyruvate oxidation, generating hydrogen peroxide. The hydrogen peroxide produced by this enzymatic reaction is then oxidized at the electrode surface, producing an electrical current. The amount of current generated is proportional to the concentration of hydrogen peroxide, which in turn is proportional to the lactate concentration in the sample.

**Figure 5:**
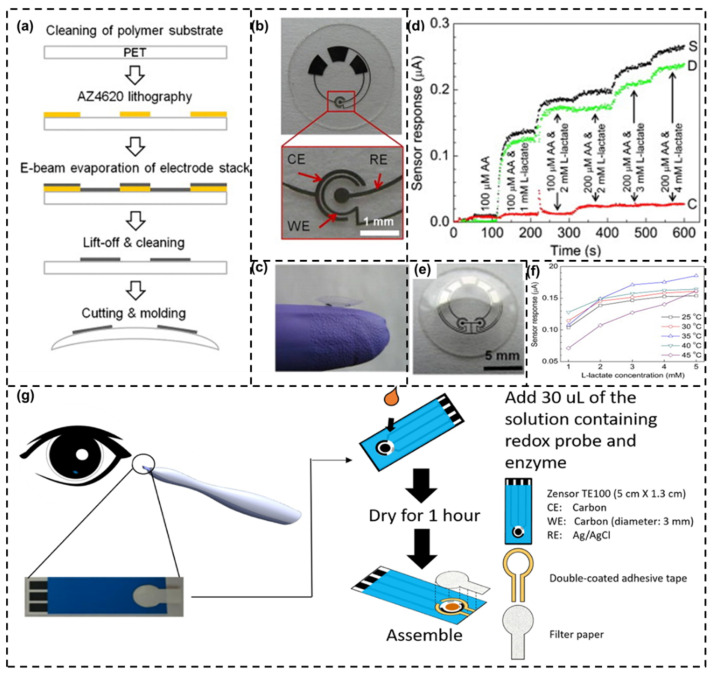
(**a**) Fabrication process of l-lactate sensor on PET substrate molded into a contact-lens shape, (**b**) flat substrate comprising of sensing unit, interconnects, and electrodes connected to external potentiostat (CE—counter electrode, WE—working electrode, and RE—reference electrode), (**c**) fabricated contact lens sensor, (**d**) current response over time for dual sensor setup (S—functionalized sensor, C—control sensor, and D—differential signal), (**e**) optical image of a lens with the dual sensors configuration, (**f**) temperature stability of l-lactate sensor where current measurement was performed against l-lactate concentration for a varying temperature of 20–45 °C (reprinted with permission from [100]), and (**g**) tear lactate sensor fabrication and assembly. Tear lactate test strip inserts to a pen-like meter collects tear in contact with conjunctiva using a filter paper. Sensor comprised of carbon working electrode (WE), carbon counter electrode (CE), and silver/silver chloride (Ag/AgCl) reference electrode (RE) (Reprinted with permission from [158]).

An electronic enzymatic l-lactate sensor was fabricated for the monitoring of l-lactate levels in tear by Thomas et al. [100]. The sensor was made from a polymer substrate, namely poly (ethylene terepthalate) (PET), which was lithographically patterned using AZ 4620 positive resist and molded into a contact lens shape [Figure 5a–c]. Here, LO_x_ was immobilized on Pt sensing electrodes with glutaraldehyde and bovine serum albumin. The sensor exhibited a quick response time of 35 s and an average sensitivity of ~53 μAmM^−1^ cm^−2^, both within their linear range. Moreover, a dual sensor to evaluate the current response (Figure 5d,e) by measuring a differential current signal proportional to l-lactate for suppressing interfering signals was designed. The sensors were functional at a temperature relative to that at the eye surface and their response was found to be stable up to 24 h (evident from Figure 5f). A lactate sensitive potentiometric sensor based on polypyrrole films displayed a good value of recovery during measuring lactate values in tear [157]. Oxygen deficiency and fluctuation often affect lactate oxidation and could introduce errors in measured lactate concentration. To circumvent these limitations, a tear-based sensor was developed with the aid of a protein engineered LOx and a Schirmer’s test strip [158]. The sensor was developed based on a redox solution constituting the engineered LOx and an innovative tear sampling component made of a Schirmer’s test strip that was affixed to absorb simulated tear fluid samples. The sensor fabrication is depicted in Figure 5g. With a detection range of 0.39–16.60 mM and shelf life of a minimum of 8 weeks, the sensor was not found to be interfered with by the presence of acetaminophen, ascorbic acid, and uric acid. Another method is to use lactate dehydrogenase (LDH) enzyme in combination with a dye-based system as an optical biosensor. In this method, the enzyme LDH catalyzes the reaction of lactate with a coenzyme such as nicotinamide adenine dinucleotide (NAD^+^), resulting in a change in color or fluorescence that can be measured optically. Recent advancements in wearable technology are enabling the development of devices such as smart contact lenses or patches that can continuously monitor lactate levels in tears. These devices are designed to be comfortable and unobtrusive.

### 4.4. Proteins, Lipids and Electrolyte Monitoring

Apart from the use of major metabolites such as glucose and lactate, other tear biomarkers, including proteins, lipids, and electrolytes, are also being explored for sensing applications. Proteins in tear fluid control wound healing, inflammatory responses, and antibacterial protection [61,159]. Moreddu et al. [147] developed a microfluidic contact lens-based wearable tear sensor for in situ tear protein sensing using artificial tears. The microfluidic system was inscribed in commercial contact lenses and a colorimetric biochemical protein sensor was deposited over the sensing area. When the tear gets bound, the sensor exhibits a reflection peak shift in the visible spectrum, resulting in a color change (a light blue color) corresponding to a concentration of 5 g/L. Biosensors were later imaged using a smartphone camera and further processed using a MATLAB algorithm, which provided the concentration of analytes corresponding to the detected color. The developed biosensor responded within a time range of 15 s and yielded a LOD of 0.63 g/L and a sensitivity of 0.49 nm/g L^−1^ of proteins. This sensor has proved the potential to aid in the diagnosis and monitoring of diseases such as rosacea, diabetes, keratoconus, and uveitis. Subsequently, Xu et al. [151] developed a novel non-invasive wearable eye patch biosensor for detection of protein in tears. Here, a drop of tear infiltrates the eye patch within 2 s to reach the sensing area occupied with specific colorimetric assays (sodium citrate buffer and tetrabromophenol blue). This results in blue color signals from the sensing region on the eye patch that are captured and analyzed using a smartphone to achieve rapid and accurate detection of protein. For sensing the protein, the optimal concentration of assay used was 0.25% within a reaction time of 2 s and LOD was found to be 17 g/L. These developed protein biosensors facilitate the widespread application of tear-based noninvasive wearable biosensor in emerging personal health care and clinical diagnosis.

Tear lipid layer plays an important role in maintaining ocular surface homeostasis. As mentioned in Section 3.2, analytical lipidomics presents several challenges owing to the limited quantity of unstimulated tears, which hampers widespread research in the field of lipid-based biosensors. Song et al. [160] developed a contact lens-based smart wearable cholesterol biosensor for selective detection of free cholesterol to monitor hyperlipidemia (high cholesterol) patients using a smartphone. Here, the contact lens was embedded by integrating an electrochemical cholesterol biosensor, a stretchable wireless antenna, and an integrated circuit (IC) chip. Cholesterol oxidase enzyme was used for detecting cholesterol in tears. The underlying mechanism is based on the cholesterol oxidation catalyzed by the contact of immobilized cholesterol oxidase and free cholesterol to form cholest-4-en-3-one and hydrogen peroxide. Later, a wireless communication system with a flexible antenna and an IC chip was established for enabling the smartphone for transferring power and data in a wireless manner. The measured cholesterol range was shown on the screen of a smartphone. This is the first reported cholesterol monitoring healthcare device using tear fluid, and it demonstrated positive correlation with hyperlipidemia in a rabbit model, paving the way for future clinical human studies.

Tear electrolytes are promising target analytes to assist wearable devices to measure tear osmolarity in a clinical perspective. A fluorescence-based contact lens biosensor with the aid of fluorophores was developed to measure Na^+^ and Cl^−^ ions concentration in tear for measuring the hydration status of human body [161]. These fluorophores, which are sensitive to Na^+^ and Cl^−^ ions, showed spectral variation in the physiological range for Na^+^ and Cl^−^ ions in tears. These findings proved the ability of such contact lenses to be used for rapid non-invasive detection of whole-body hydration and associated health issues. A paper-based microfluidic system coupled with a smartphone was reported by Yetisen et al. [162] to detect the ionic concentration of Na^+^, K^+^, and Ca^2+^ in artificial tear solution for diagnosing dry eye at point-of-care settings. The sensing regions were functionalized with fluorescent chelating agents, which are specific to electrolytes, and their fluorescence outputs were measured using a smartphone. This device highlights the practical feasibility and effectiveness for dry eye diagnostics. In addition to these, holographic nanostructures generated by direct laser writing on contact lenses detected electrolyte concentration changes in tear fluid composition [163]. Relevant tear-based biosensors are summarized in Table 1.

## 5. Outlook and Conclusions

Similar to other wearable biosensor technologies, there exist several technical challenges associated with developing and deploying tear-based biosensors, such as miniaturization, power consumption, and long-term stability. Miniaturization is necessary for comfortable wear and integration into contact lenses. Integration of miniaturized multiple sensor components such as electrodes, transducers, and fluidic channels needs advanced materials and fabrication technologies. On the other hand, tiny sensors produce weak signals, which need further amplification and processing. Such challenges can be overcome by utilizing advancements in the field of 3D printing and photolithography for the sensor to be miniaturized without compromising its efficacy [110]. Utilizing 2D metal organic frameworks (MOFs), transition metal dichalcogenides and nanomaterials such as carbon nanotubes, graphene, etc., can increase the sensitivity while reducing the size of the sensor components [127]. Although miniaturization is necessary for wearability, it must be balanced with the need for reliable sensors. Owing to the small form-factor as well as portability and conformability aspects, similar to other wearable biosensors, deficient power supply is a limiting factor hampering the development in this research direction. Low-power electronics, energy harvesting, and intelligent power management algorithms are techniques being implemented to maximize the power efficiency to improve battery life [168]. Practical, durable, and eco-friendly energy harvesting techniques, which include solar, thermal, radio frequency energy, kinetic energy, and biomass energy, are garnering significant research attention in this regard [169]. In addition, the long-term stability of tear-based sensors depends on the ocular environment or contact with tear drops, which may induce biofouling and chemical and mechanical degradation subject to varying disease conditions, pH, and temperature [170].

The complex nature of tear fluid, along with lower levels of biomarkers, varying tear composition during emotion and irritation, tear sample evaporation, and variations in tear generation with individuals, render significant challenges to research in this direction. The development of sensors to detect a broad spectrum of biomarker candidates needs to be researched in detail, including novel bio-affinity sensors [171]. For instance, exosomes in tears have been used for non-invasive breast cancer detection using TearExo^®^, which is based on a fluorescent exosome sensing chip and an automatic exosome analyzer [172]. Tear sample collection using techniques such as Schirmer’s strips need extra precaution as there is a chance of damage to the ocular surface due to contact with conjunctiva, which can induce eye irritations. On the contrary, capillary based techniques, despite being less invasive, are often a slow method, collect low sample volumes (particularly unsuitable for DED), and pose difficulty when trying to collect tear from pediatric patients [159]. Most importantly, the problems associated with high intra-individual variability and “pre-analytical variability” associated with the removal and handling of tear fluids need to be examined [173].

Reduced sample volume, lower glucose levels, and evaporation issues of tear fluid pose correlation issues with blood glucose level [174]. It is essential to develop prospective technologies to evaluate glucose level fluctuations towards detecting hyperglycemic events to provide technologies for interstitial glucose analysis, which is still challenging [119]. As mentioned in Section 4.2, pH sensing of tear fluids is often affected by a change in the color of chromogenic reagents when coming in contact with the target biomolecules or ions. Another concern is associated with the influence of ambient light on the collected color data and respective measured concentration values [175]. Tear sensors are often used in conjunction with smartphones for improved detection of analytes of interest. There are limitations associated with correlating tear glucose with blood glucose levels owing to the alteration in tear compositions brought about by the physical stimulation of the eye. Hence, the focus on wearable sensors should be shifted towards developing materials and designs capable of minimizing tear stimulations when worn. For instance, the concept of ‘personalized lag time’ introduced by Park et al. [164] expands the technique to analyze biomarkers in other body fluids (blood, sweat) to integrate other wearable technologies such as smart watches and fitness trackers.

Advanced materials systems developed for such wearable tear sensors should ensure eye comfort, should be stretchable to conform to the complex movements, and should not induce any temperature rise due to the energy generated during wireless transmission, which is capable of potentially harming eyes [128]. Associated complex design of such advanced materials with ultra-precision for improved performance requires high-end specialized fabrication machines that can pose economic constraints to several end-users [176]. In addition, the fabrication and disposal of such sophisticated materials needs to be designed in a sustained way to alleviate the environmental impact. Development of tear-based wearable technologies needs to acquire approval from regulatory bodies such as the International Electrochemical Commission (IEC) and International Organization for Standardisation (ISO) standards (IEC 62304, IEC 60601, ISO 14971, and ISO 13485) in relation to design and clinical validation of wearable sensor systems [177]. FDA guidelines need to be met, especially those pertaining to wearable medical devices in terms of risk classification, as well as various premarket submissions, such as the 510(k) pathway, De Novo pathway, and premarket approval applications [178].

More clinical studies that consider the implementation aspects, toxicity, and immunogenicity of the tear based wearable sensors need to be conducted. Continuous analyte monitoring using tear-based wearable sensors to predict and prevent various disease conditions need a multidisciplinary approach by chemists, biologists, engineers, and clinicians. Integration of deep learning techniques, which provide innovative technologies in data acquisition systems along with efficient data-fusion and data-mining methods, could enable innovative tear-based point-of-care technologies beneficial for personalized healthcare. Such technologies can be a double-edged sword due to the vulnerability of such technologies to cyber-attacks or other intrusive events and hence techniques to assure data protection [179] must be considered. The vast amount of human research data collected with wearable sensing technologies pose serious concerns associated with data privacy and security. Owing to the multidisciplinary aspects of wearable technologies, there are many possible hindrances, including ethical guidelines that may vary from fitness tracking sensors to medical grade sensors. The research community and regulatory bodies need to establish frameworks and specific guidelines for all stakeholders when it comes to addressing various ethical concerns, risk assessing, data privacy, and consent [180].

Continuous improvement in material technologies, miniaturization aspects, tear sample collection, power harvesting, and wireless systems integrated with potential AI algorithms will pave the way for futuristic tear-based wearable sensing technologies. Overall, the present review summarizes recent advancements in the field of tear-based ocular biosensors for potential wearable technologies. The relevance of specific tear biomarkers such as proteins, lipids, electrolytes, and metabolites are pinpointed to monitor and diagnose various ocular diseases. Specific sensing technologies based on tear biomarkers are comprehensively reviewed, with a focus on their underlying mechanisms. Most importantly, several limiting factors hampering the development of potential tear-based biosensors are covered to assist future research in the field of tear-based point of care medical devices.

## Figures and Tables

**Table 1:** Developed tear-based biosensors for human healthcare monitoring.

Biomarker	Biorecognition Molecule	Sensing Mode	Type of Biosensors	Working Range	Sensitivity	Limit of Detection (LOD)	Ref
Protein (Lactoferrin)	Terbium chloride	Fluorescence	Contact lens	0–5 mg/mL	-	0.44 mg/mL	[78]
Glucose	Glucose oxidase	Electrochemical	Contact lens	0.18–0.7 mM	1% current change per 0.047 mM	0.02 mM	[164]
Electrolyte (Na^+^)	Fluorescent diaza-15-crown-5	Fluorescence	Strip-based	130–150 mmol/L	2.7 mmol/L	1 mM/L (avg detection error)	[162]
Electrolyte (K^+^)	Fluorescent diaza-15-crown-6	Fluorescence	Strip-based	24–26 mmol/L	1.4 mmol/L	1.3 mM/L (avg detection error)	[162]
Glucose	Boronic acid-PVA hydrogel	Colorimetric	Contact lens	0–50 mM	-	0.05 mM	[124]
Protein	3′,3′,5′,5′-tetrachlorophenol-3,4,5,6-tetrabromosulfophthalein	Colorimetric	Contact lens	0.5–5 g/L	0.49 nm/gL^−1^	0.63 g/L	[147]
Glucose	Glucose oxidase	Electrochemical	Contact lens	0–12 mM	9.7 μA mM^–1^ cm^–2^.	9.5 μM	[165]
Cholesterol	Cholesterol oxidase	Electrochemical	Contact lens	0.4–46.4 mg/dL	1% current change per 0.0043 mM	0.38 mg/dL	[160]
Electrolyte (Na^+^)	sodium green-poly-l-lysine	Fluorescence	Contact lens	0–120 mM	-	0.2 mM	[161]
Electrolyte (Cl^−^)	OD-MQB fluorophore of 6-methoxyquinoline and 1-bromooctadecane	Fluorescence	Contact lens	0–120 mM	-	10 mM	[161]
Glucose	γ-Fe_2_O_3_@NiO magnetic oxide nanosheets	Electrochemical	Contact lens	0.005–6.0 mM	0.17 MHz mmHg^−1^	0.43 μmol	[141]
Glucose	Glucose oxidase/peroxidase/3,3′,5,5′-tetramethylbenzidine	Colorimetric	Contact lens	0–20 mM/L	1.4 nm/mmol L^−1^	1.84 mmol/L	[147]
l-Lactate	lactase oxidase	Electrochemical	Contact lens	1–5 mM	53 μA mM^−1^ cm^−2^	1.75 mM	[100]
Glucose	3,3′,5,5′-tetramethylbenzydine	Colorimetric	Microcapillary tube	0.1–1 mM	84 AU/mM	50 µM	[166]
Protein	Matrix metalloproteinase-9	Electrochemical	contact lens	1–500 ng/mL	11.1 ng/mL per 1% of change in drain current	0.74 ng/mL	[167]
